# Single fiber reflectance spectroscopy for pancreatic cancer detection during endoscopic ultrasound guided fine needle biopsy: a prospective cohort study

**DOI:** 10.7150/ijms.65364

**Published:** 2022-01-01

**Authors:** Labrinus van Manen, Iris Schmidt, Akin Inderson, Ruben D. Houvast, Jurjen J. Boonstra, Jouke Dijkstra, Jeanin E. van Hooft, Wouter B. Nagengast, Dominic J. Robinson, Alexander L. Vahrmeijer, J. Sven D. Mieog

**Affiliations:** 1 Department of Surgery, Leiden University Medical Center, Leiden, The Netherlands.; 2 Department of Gastroenterology and Hepatology, University of Groningen, University Medical Center Groningen, Groningen, The Netherlands.; 3 Department of Gastroenterology and Hepatology, Leiden University Medical Center, Leiden, The Netherlands.; 4 Division of Image Processing, Department of Radiology, Leiden University Medical Center, Leiden, The Netherlands.; 5 Department of Otorhinolaryngology and Head and Neck Surgery, Center for Optical Diagnostics and Therapy, Erasmus Medical Center, Rotterdam, The Netherlands.

**Keywords:** pancreatic cancer, diagnosis, spectroscopy, endoscopic ultrasound, fine needle biopsy.

## Abstract

This study aimed to determine the ability of single fiber reflectance (SFR) spectroscopy incorporated in endoscopic ultrasound fine needle biopsy (EUS-FNB) procedures in the pancreas to distinguish benign and malignant pancreatic tissue in patient with pancreatic masses suspected for malignancy.

**Methods:** This study was designed as a prospective observational single center study and included consecutive adult patients, who were scheduled for EUS-FNB of a solid pancreatic mass suspected for pancreatic ductal adenocarcinoma (PDAC). In total, seven optical parameters, derived from the absorption acquired spectra, were analyzed: blood volume fraction (BVF), microvascular saturation, average vessel diameter, bilirubin concentration (BIL), Mie amplitude, Mie slope and Rayleigh amplitude.

**Results:** Forty-five patients with a suspicious pancreatic lesion undergoing EUS-FNB were included, of which most of the patients (N=34) were ultimately diagnosed with PDAC. Finally, 27 out of 45 (60.0%) patients were used for the final analysis of the optical parameters. The median (IQR) BVF differed significantly in benign compared to malignant tissue (0.86 [0.30-2.03] and 4.49 [1.28-15.47]; *p*=0.046). Combining BVF and BIL to a new parameter (*θ*) improved the discrimination between PDAC and benign pancreatic tissue (*p*=0.026). The area under the curve of *θ* was 0.84, resulting in a 92.8%, 75.0%, 97.5%, 50.0% and 91.3% sensitivity, specificity, positive predictive value, negative predictive value and diagnostic accuracy for detection of PDAC.

**Conclusion:** Differentiation between PDAC and benign pancreatic tissue using SFR spectroscopy during EUS-FNB procedures is promising. Future work should focus on comparing the diagnostic performance combining SFR spectroscopy with EUS-FNB and EUS-FNB alone.

## Introduction

Correct identification of pancreatic ductal adenocarcinoma (PDAC) can be difficult as it shows a considerable overlap with benign diseases like fibrosis or pancreatitis 1. Discrimination between benign and malignant disease is of paramount importance in order to apply the most optimal treatment strategy. Next to conventional radiological imaging obtaining tissue is the cornerstone during the diagnostic process. Endoscopic ultrasound fine needle aspiration (EUS-FNA) or fine needle biopsy (EUS-FNB) are considered the least invasive and most effective procedures for establishing the diagnosis of pancreatic tumors. It has been suggested that EUS-FNA has some limitations such as the need of multiple tissue punctures ('passes') and preferably rapid onsite evaluation 2-4. The introduction of fine needle biopsy in the endoscopy theatre, enabling acquisition of tissue cores, resulted in an improved diagnostic accuracy (between 85% and 95%), significantly lower number of passes, and similar diagnostic adequacy (between 87% and 96%) compared to EUS-FNA 5-7. The presence of chronic pancreatitis is one of the factors mainly affecting the accuracy of both EUS-FNA and FNB, indicating that discriminating pseudotumoral masses from PDAC in the setting of chronic pancreatitis is of paramount importance in order to increase the diagnostic yield 8, 9. Therefore innovative techniques, such as contrast-enhanced EUS-FNA 10 or EUS elastography 11, are currently being investigated to determine the additional value during EUS in order to improve the differentiation between PDAC and pancreatitis or normal pancreatic tissue.

Multi-diameter single fiber reflectance spectroscopy is such an innovative technique that enables the non-invasive quantification of absorption and scattering parameters of tissue. This technique combines the data from two single fiber reflectance (SFR) spectra acquired with two different fiber diameters. Tissue characterization based on the quantification of physiological parameters, such as the blood volume fraction and microvascular oxygen saturation has the potential to differentiate between malignant and benign tissues directly during endoscopy. To extract and quantify these parameters from the obtained spectra a validated mathematical model, based on the knowledge of the absorption spectra of the chromophores, is applied 12, 13. However, this method can only measure superficial tissue and not in depth as is needed with pancreatic tissue. SFR spectroscopy using only one fiber diameter solves this problem as it reduces the diameter of the fiber. This fiber can be guided through a 22G needle used during EUS and measure at the same position, during the same needle pass as EUS-FNB is performed within the pancreatic mass 14. Our group previously found a correlation between the acquired absorption spectra and cytology in a small group of patients with a variety of included pancreatic benign and malignant lesions 14.

In this study the ability of SFR spectroscopy incorporated in EUS-FNB procedures of the pancreas to distinguish benign and malignant pancreatic tissue has been assessed in a larger patient cohort with optimised data exclusion procedures.

## Material and methods

### Study design

This study was designed as a prospective intention-to-measure single center trial. Consecutive adult patients, who were scheduled for EUS-FNB of a solid pancreatic mass suspected for carcinoma at the Leiden University Medical Center were eligible for inclusion, unless they refused participation or if insufficient time (≤ 3 days) was available to consider participation. Patients with cystic pancreatic lesions (either non-invasive intraductal papillary mucinous neoplasm, mucinous cystic neoplasm and serous cystadenoma) or a (suspicion of) neuro-endocrine tumor were not included. This trial was approved by the local Institutional Review Board (P17.316) and was performed in accordance with the ethical standards of the Helsinki Declaration of 1975. This trial was registered at the Dutch Trial Register (NL7613). All patients provided informed consent.

### Endoscopic procedure

**Figure 1:**
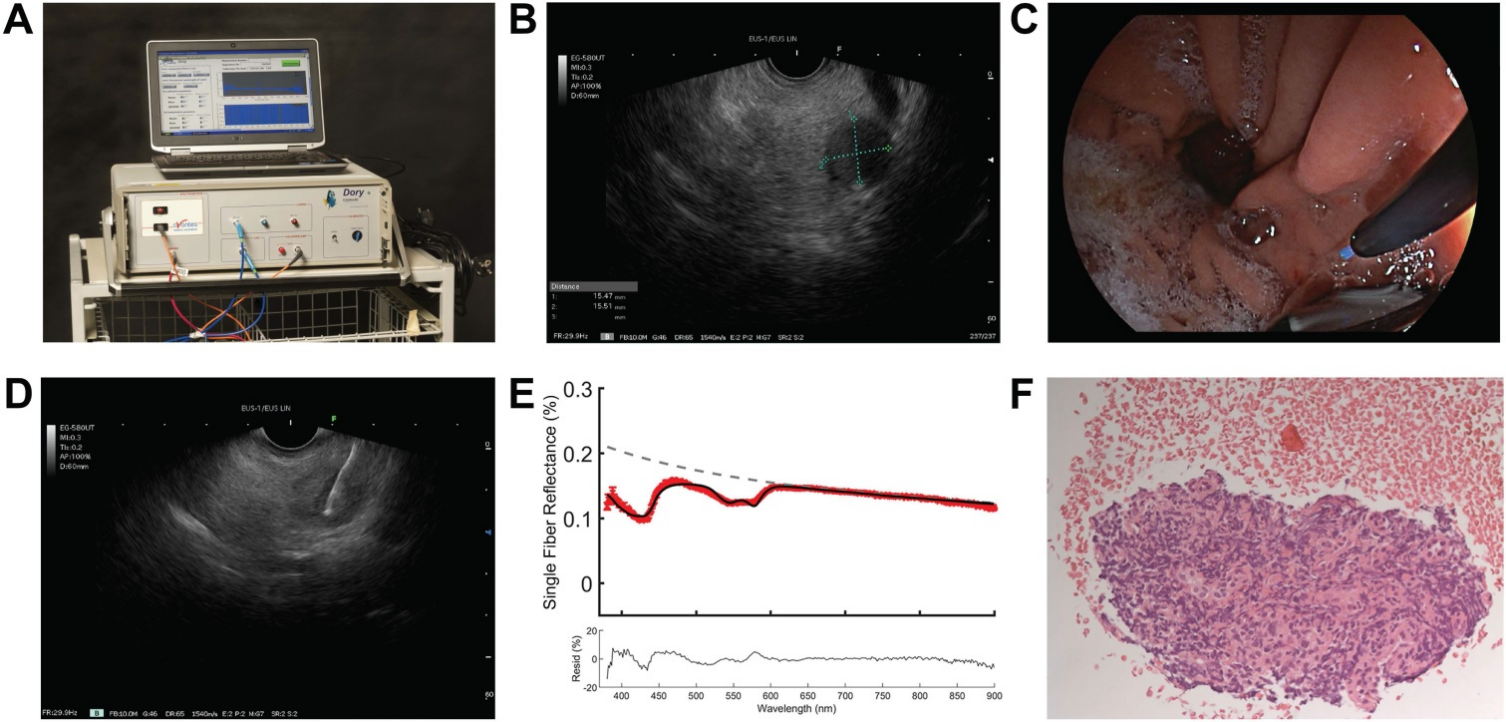
** Overview on implementation of SFR spectroscopy during endoscopic procedure.** (**A**) Overview of spectroscopy device. (**B**) identification of pancreatic mass using endoscopic ultrasound. (**C**) insertion of the optical fiber under white light endoscopic guidance (**D**) performing spectroscopic measurement in the suspicious lesion under endoscopic ultrasound guidance. (**E**) representative reflectance spectra of a malignant lesion. (**F**) histological confirmation of malignant cells in the acquired tissue.

The EUS procedure was performed with the patient positioned in left lateral decubitus position under conscious (midazolam) or deep sedation (propofol). After introduction of a curved linear array echo endoscope (Fujinon EG-580UT, Fujifilm Corporation, Tokyo, Japan), visualization of the pancreatic mass, and decision to take a biopsy, a sterile 22-gauge biopsy needle (Acquire EUS FNB Needle, Boston Scientific, Marlborough, USA) was inserted in the endoscopic working channel. The stylet of the needle was replaced by a sterilized optical fiber (Light Guide Optics, Germany), which was placed in the pancreatic mass under ultrasound guidance (Figure 1). A minimum of three spectroscopic measurements were performed on the intended biopsy location before biopsies were taken. After each pass, the obtained material was directly placed into formalin and the adequacy of the samples was examined on-site by the endoscopist. In general, two passes were needed to obtain sufficient amount of tissue. For each biopsy area, the spectroscopic measurement procedure was repeated as described if feasible during the procedure. After each procedure, the spectroscopic fiber was calibrated in 2% Intralipid 20% and a dark container with water. The acquired tissue samples were paraffin embedded, cut into sections and stained using hematoxylin and eosin. If necessary, additional immunohistochemical staining was performed. All histology sections were examined by an experienced pancreatic pathologist for final histologic diagnosis.

### SFR spectroscopy

The spectroscopy setup used in this study has been previously described in more detail 14. In brief, the setup consists of a spectrophotometer (SD-2000; Ocean Optics, The Netherlands) to measure the white light reflectance, a halogen light source (HL-2000-FHSA; Ocean Optics, The Netherlands), and a quadfurcated optical fiber that is connected to a single optical fiber (Light Guide Optics, Germany). Sterilized single-use fibers had a core diameter of 230 μm, an outer diameter of 400 μm, a SMA905 connector, a distal fiber tip polished at 5°, with a numerical aperture of 0.27, and a length of 3 m (± 0.1 m). All measurements were analysed using a previously described fitting procedure 14. The absorption coefficient of the measured spectra is modelled using a modified Beer-Lambert law. An assumption is made for the reduced scattering coefficient (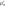) and phase function (PF) at one wavelength, with wavelength dependent changes estimated by a background scattering model as there is no a priori knowledge of these parameters 15, 16. Next, a Levenberg-Marquardt algorithm was applied to extract parameter values and the confidence interval was calculated from the square root of the diagonal of the covariance matrix. In total, seven optical parameters were evaluated for the differentiation between malignant and benign tissue: blood volume fraction (BVF), microvascular saturation parameter (StO_2_), vessel diameter (VD), bilirubin concentration (BIL), Mie amplitude, Mie slope and Rayleigh amplitude. The first four parameters are physiological (absorption) parameters and the last three are morphological (scattering) tissue parameters.

### Exclusion criteria SFR data

Spectra were evaluated by applying four predefined rejection criteria, which were developed before the data analysis by two members of our study team (IS and DR), who were not involved in the clinical procedures: (1) non-convergence of model fit, (2) absolute residual > 25% (between 450-900 nm) and (3) confidence interval (CI) which was >100x higher as the mean of the remaining CI parameters using the same calibration procedure (4) blood volume fraction higher than 40% indicating a blood pool. For the non-convergence fit, the fit did not converge due to imaginary numbers and values were set to not-a-number. The absolute residual is increased when a systematic error in the fitting model is present. A low absolute value, below 25%, therefore indicates the goodness of the fitting procedure. A high CI shows the statistical error of a parameter. A high CI indicates a low signal to noise ratio for the measurement. Finally, blood pool causes measurements of blood instead of the tissue we are interested in 14.

### Power calculation and statistical analysis

A sample size calculation using A'Hern's single stage phase II trial design with alpha=0.05 and power=80%, showed that 45 patients are needed to distinguish between a detected pancreatic mass by SFR spectroscopy of 90% and 75% or less (unacceptable outcome) 17. This requires at least 39 patients with biopsies positive for PDAC and six with biopsies negative for PDAC to reach the positive endpoint (i.e. diagnostic accuracy of 90%), which is concordance with the diagnostic yield of EUS-FNB 2.

The optical parameters were calculated by averaging repeated measurements of the same location per patient weighted by the individual fit confidence intervals. Continuous variables were presented as mean values including standard deviation (SD) in normal distributed data or as median values including interquartile range (IQR) in non-normal distributed data, respectively. Differences between two groups were analyzed using the Mann-Whitney U test (non-normally distributed data). The most clinically relevant optical parameters and serum bilirubin were further evaluated in a multivariate binary logistic regression model to investigate to effect on differentiation between malignant and benign tissue. Data was standardized to a normal distribution 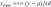, where µ is the mean and sd the standard deviation of parameter 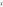. Outliers were identified using the ROUT method 18. Using a linear discriminant analysis, variables with a *p*-value below 0.200 were combined to create a new variable *θ*. Receiver-operating characteristic curve analyses were used, after which the sensitivity, specificity, positive predictive value (PPV), negative predictive value (NPV), and diagnostic accuracy were calculated. A *p*-value below 0.05 (two-sided) was considered statistically significant. Statistical analysis was performed with SPSS statistical software (Version 25.0, Chicago, Illinois) and graphs were created with Graphpad 9.0 (GraphPad Software, Inc., San Diego, CA).

## Results

### Patient characteristics

**Figure 2:**
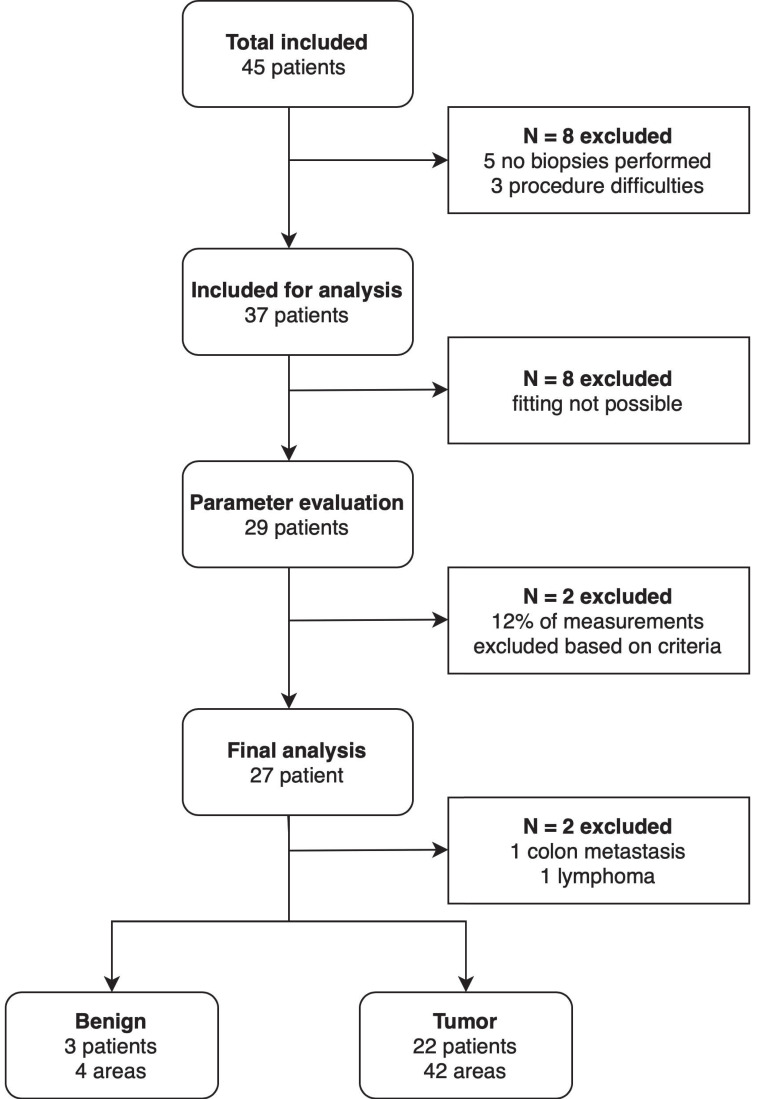
Flowchart of the included patients.

Forty-five patients with a suspicious lesion in the pancreas undergoing EUS-FNB were included in this study, of which two-thirds (N=30) had a lesion in the pancreatic head (Table 1). Thirty-four (75.6%) were diagnosed with PDAC. In total, 11 (24.4%) of the included patients underwent surgery (Table 1). Figure 2 shows an overview of all in- and excluded data. Thirty-seven out of the 45 patients were included for analysis. Reasons for exclusion were not performing biopsies during the procedure (N=5) and difficulties during the endoscopic procedure interfering spectroscopic measurements (N=3). For eight patients the fitting procedure was not possible due to an incorrect calibration procedure, resulting in 29 patients in which optical parameter evaluation was possible. Of the remaining 29 patients, 12% of the measurements were rejected based on the previously mentioned exclusion criteria. This resulted in the exclusion of two additional patients as all measurements of these patients were rejected. Ultimately, 27 patients were used for the final analysis of the optical parameters. In two patients the pancreatic mass was found out to be a colon carcinoma metastasis and a lymphoma, respectively. Four benign areas were measured in three patients and malignant tissue was measured in 42 areas in 22 patients. None of the patients had measurements of both benign and malignant tissue.

### SFR spectra and optical parameters

**Figure 3:**
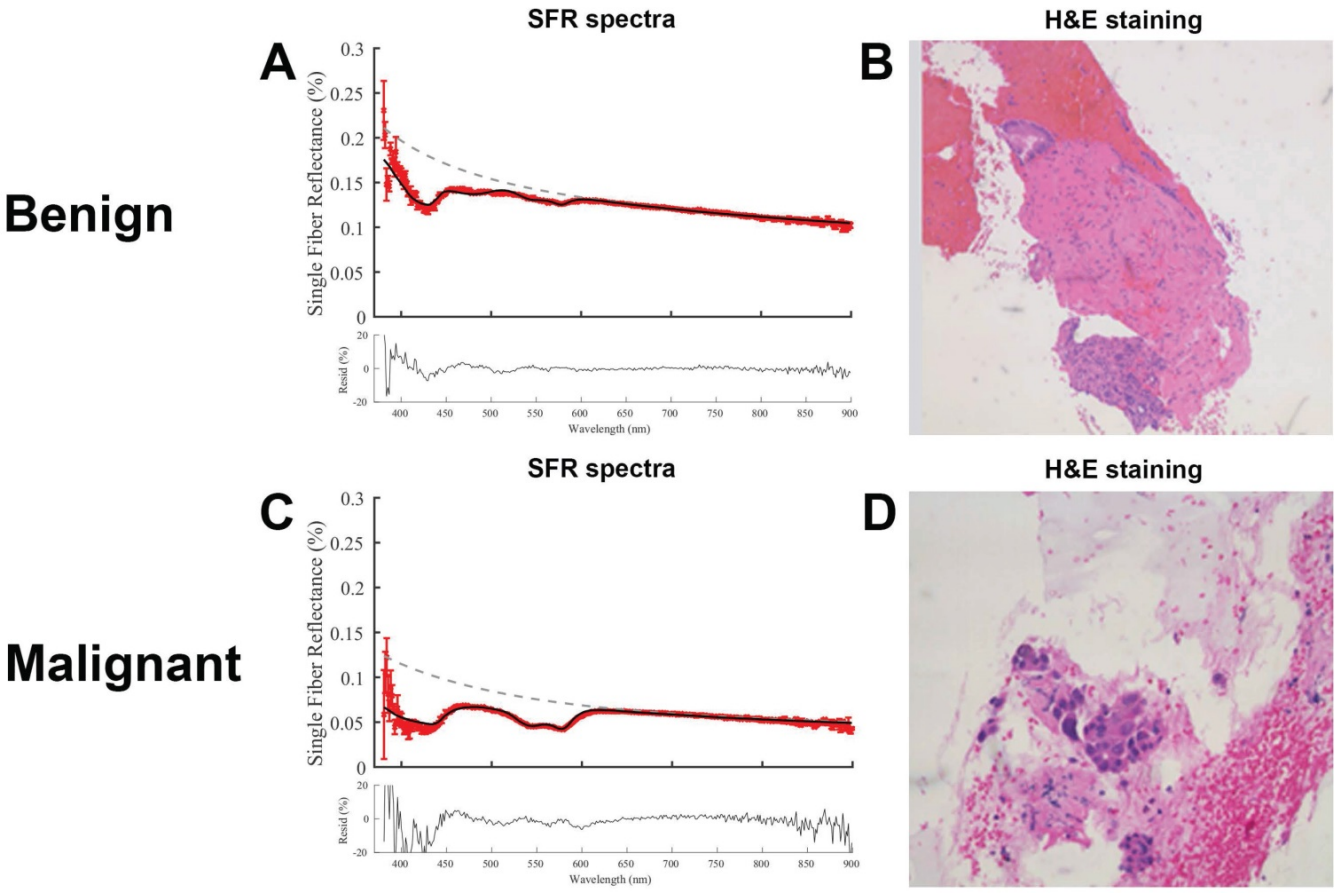
** Representative fitted spectra with corresponding histology.** (**A**) Spectra of benign lesion in the pancreas and (**B**) corresponding hematoxylin and eosin (H&E) image. (**C**) Spectra of a malignant pancreatic lesion and (d) corresponding H&E image.

Representative SFR spectra for both benign and malignant tissue including the corresponding histology are shown in Figure 3. Both residuals show low noise suggesting a correct fitting model. The malignant tissue shows a higher absorption between 500-600 nm, compared to the benign tissue, that is consistent with a higher blood volume fraction.

**Figure 4:**
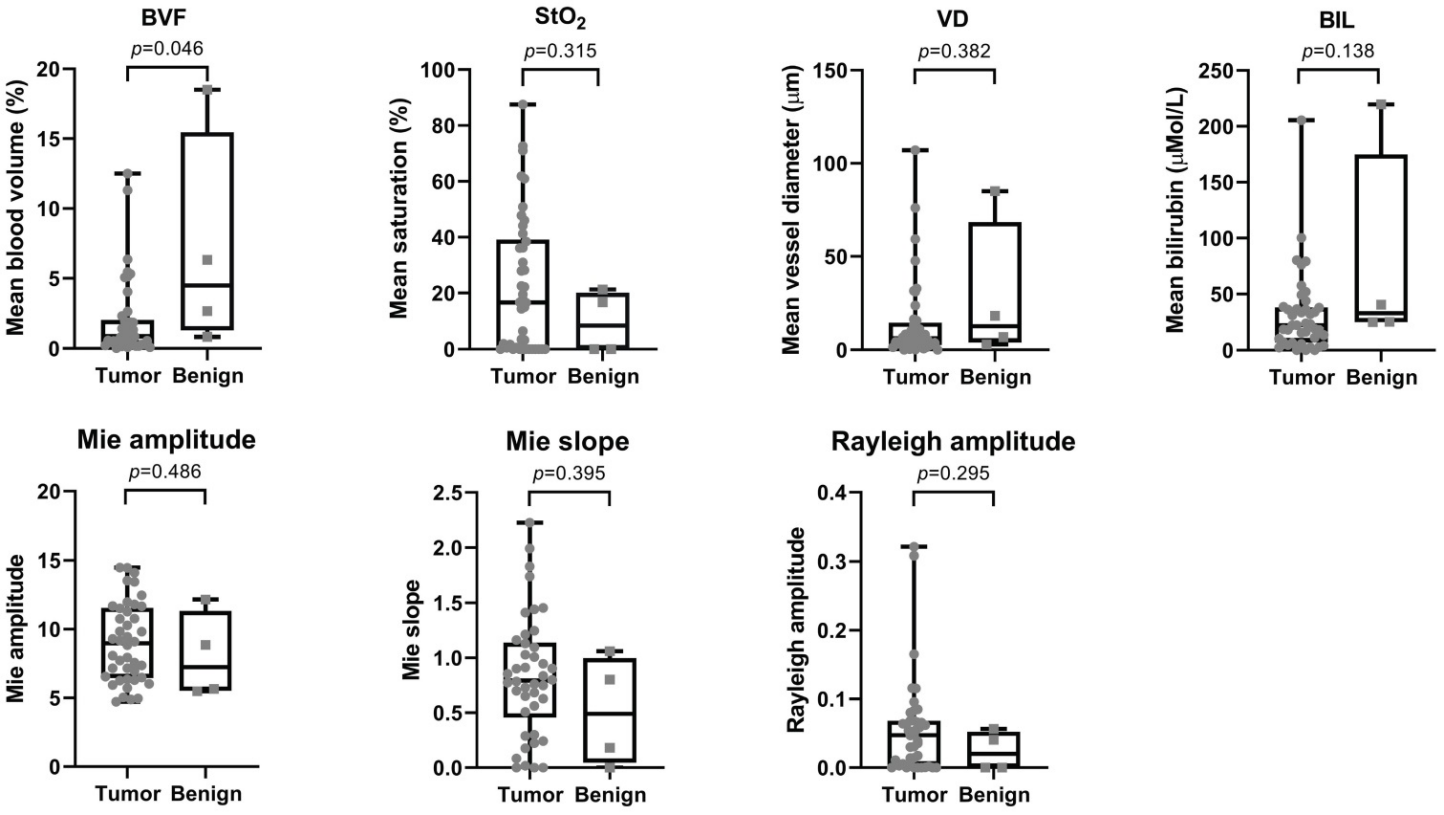
** Overview of optical properties for both tumor and benign tissue.** The top row shows the scattering properties and the bottom row the absorption properties. *P*-values are displayed above each graph.

Table 2 shows the median and interquartile range (IQR) for all analyzed optical parameters for both malignant and benign tissue. BVF and BIL were the two most significant parameters (*p*=0.046; *p*=0.138) with a median (IQR) of 0.86 (0.30-2.03) and 22.2 (6.92-38.07) for malignant and 4.49 (1.28-15.47) and 32.9 (24.90-175.00) for benign tissue, respectively (Figure 4).

### Sensitivity analysis

Within the BVF values, seven outliers were identified. To determine the effect of these outliers, the blood volume exclusion criteria was reduced from 40 to 10%. This reduction resulted in 1.3% extra measurements rejected, but still resulted in six new outliers. Furthermore, it did not show an improvement in differentiation between benign and malignant tissue (Table 2). Therefore, the exclusion criteria were not adjusted based on these results.

A binomial logistic regression was performed on the weighted mean to determine the effect on the serum bilirubin, physiological and morphological parameters on the discrimination between malignant and benign tissue. In the multivariate analysis, the BVF, StO_2_, BIL, and serum BIL were not statistically significant associated with presence of malignancy (Table S1).

### Combining parameters

**Figure 5:**
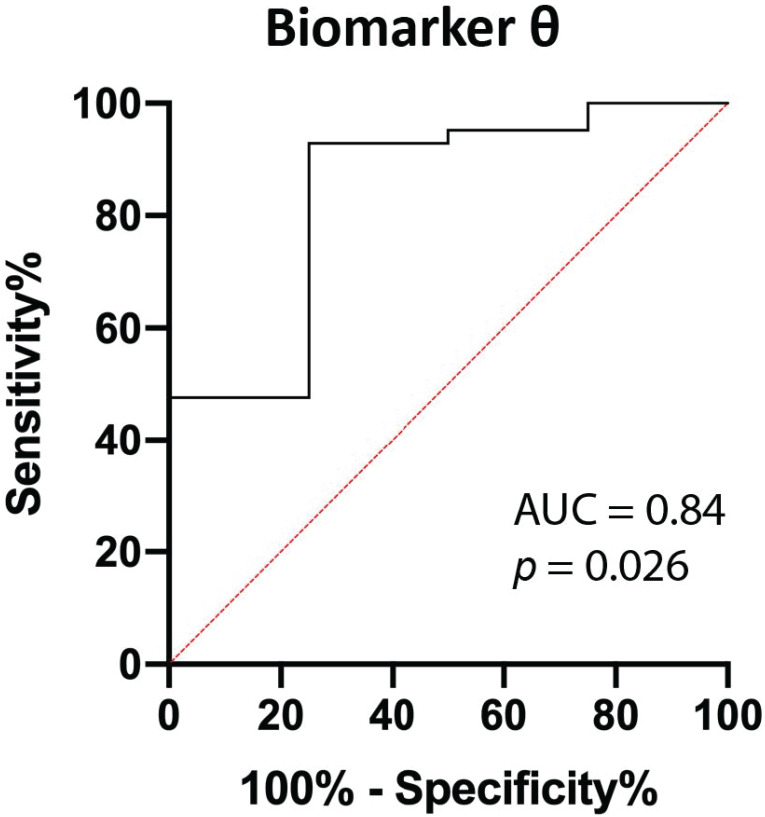
** ROC curve of biomarker *θ*.** Biomarker *θ* is composed of blood volume fraction (BVF) and bilirubin concentration (BIL). The area under the curve (AUC) is 0.84 (*p=*0.026).

Using linear discriminant analysis, the BVF and BIL were combined to a new parameter *θ*, as BVF and BIL were the most significantly different parameters (Table 2). This combined parameter improved the discrimination between PDAC and benign pancreatic tissue (*p*=0.026; Figure 5 and Figure S1). The area under the curve (AUC) of *θ* increased from 0.80 and 0.73 for BVF or BIL alone to 0.84, which results in a 92.9%, 75.0%, 97.5%, 50.0% and 91.3% sensitivity, specificity, PPV, NPV, accuracy respectively.

## Discussion

The aim of this study was to assess the ability of SFR spectroscopy to differentiate between benign pancreatic tissue and PDAC in consecutive patients with suspected PDAC, scheduled for EUS-FNB. Optical parameters were derived from the acquired spectra and consequently evaluated. A combined biomarker *θ* from the blood volume fraction and bilirubin concentration was created from SFR spectroscopy during EUS-FNB. This biomarker allowed for differentiation between malignant and benign pancreatic tissue (*p*=0.026), with an AUC of 0.84, resulting in 92.8%, 75.0%, 97.5%, 50.0% and 91.3% for sensitivity, specificity, PPV, NPV, and accuracy. These results suggest that the addition of SFR spectroscopy during EUS-FNB could potentially increase the diagnostic accuracy of such procedures.

Previously, a pilot study was performed with SFR spectroscopy during EUS-FNA procedures in the same patient population 14. This study showed that microvascular saturation (StO_2_) and bilirubin concentration were the physiological parameters that could differentiate between malignant and benign tissue most effectively. The low number of included patients, three benign and six malignant, could explain the discrepancy in results, concerning the StO_2_ and BVF. Furthermore, in two out of the six malignant lesions, other neoplastic types than PDAC were included in the former analysis. As described previously, PDAC is a hypovascular tumor as a result of impaired tumor vasculature and a decrease in angiogenesis 19. Together, this limits the blood flow and reduces the oxygen delivery to the tumor. It is suggested that the blood flow and blood volume in PDAC are lower in patients with PDAC compared to chronic pancreatitis and normal pancreatic tissue, which is in concordance with our results 20. One another study described the use of intraoperative optical spectroscopy to distinguish pancreatic cancer from normal pancreas and chronic pancreatitis. Using principal component analysis instead of deriving physiological and optical parameters from the reflectance measurements, these tissue types could be accurately identified with a sensitivity of 91%, specificity of 82%, PPV of 69% and NPV of 95%, and AUC of 0.89 21.

In our study, all reflectance measurements during FNB were histologically confirmed with tissue that was taken from the same location. This ensures that our reflectance results correspond directly to the histopathological diagnosis. Furthermore, a bigger sample size of patients was included in this study compared to the previous pilot study of Stegehuis et al. 14. Nevertheless, our study has some limitations. First, although we were able to include our powered sample size of 45 patients, a total of 27 patients could be included in the final analysis meaning that our results should be interpreted with caution. Data had to be excluded due to clinical and technical decisions. In some patients no biopsies were taken as the lesion could not be visualized or considered not to be malignant and in other patients difficulties during the endoscopic procedure interfered with the spectroscopic measurements. Furthermore, due to inaccurate calibrations a substantial number of patients had to be excluded. The frequency of this effect, which is necessary to obtain an optimal data set was not anticipated prior to designing the study. During the calibration procedure we did not attempt to feedback data on the calibration accuracy, which made it challenging for the involved clinicians to check the calibration quality as this is the first large prospective study that uses SFR spectroscopy in a clinical setting. In future trials, a calibration quality check will be implemented in the spectroscopy software. Finally, exclusion criteria also reduced the number of patients available for analysis, however the exclusion of data based on the technical criteria ensured that only optimal measurements were analyzed reducing the possibility of variance due to wrongly acquired data. All together this resulted in only a limited number of patients with benign areas making a fair comparison of optical parameters between malignant and benign tissue more difficult. Additionally, no patients had measurements of both benign and malignant tissue. For the last group this could have helped in determining the intraindividual instead of the interindividual variability in physiological parameters.

Implementation of this technique into clinical practice is relatively easy, as it is compatible with the endoscope and FNB needles that are currently used during these diagnostic procedures. According to a recent meta-analysis, that includes 18 high-quality designed randomized controlled trials, the introduction of FNB has led to a diagnostic adequacy of 86-90% and a diagnostic accuracy of 85-87% 6. The results of this study demonstrated that the addition of SFR spectroscopy could potentially increase this diagnostic accuracy, although the results of this study should be interpreted with some uncertainty because of underpowering due to the amount of excluded patients. Nevertheless, even with this number of patients we could already distinguish PDAC from benign pancreatic tissue with similar diagnostic accuracy to what has been reported for EUS-FNB. Therefore the results of this study first warrant a clinical trial comparing the diagnostic accuracy of EUS-FNB combined with SFR spectroscopy or EUS-FNB alone.

In conclusion, differentiation between PDAC and benign pancreatic tissue using SFR spectroscopy during EUS-FNB procedures is promising, however technical improvements in the software and further clinical trials are necessary to demonstrate the added value during EUS procedures of the pancreas.

## Supplementary Material

Supplementary figures and table.

## Figures and Tables

**Table 1:** Baseline patient characteristics.

Variable	
**Age (y), *mean (SD)***	66.2 (9.5)
**Sex, n (%)**	
Male	24 (53.3)
Female	21 (46.7)
**Bilirubin (μmol/L), *median (IQR)***	18 (8-210)
**(Suspected) tumor location, *n* (%)**	
Head	30 (66.7)
Body	9 (20.0)
Tail	6 (13.3)
**Preoperative tumor stadium, *n* (%)**	
Ia	2 (4.4)
Ib	3 (6.7)
IIa	3 (6.7)
IIb	7 (15.6)
III	14 (31.1)
IV	6 (13.3)
Not applicable	10 (22.2)
**Patients undergoing FNB, *n* (%)**	41 (91.1)
**Pathological diagnosis, *n* (%)**	
Pancreatic ductal adenocarcinoma	34 (75.6)
(Auto-immune) pancreatitis	3 (6.6)
Neuroendocrine tumor	1 (2.2)
Pancreatic fibrosis	1 (2.2)
Lymphoma	1 (2.2)
Benign pancreatic tissue	5 (11.1)
**Patients undergoing surgery, *n* (%)**	11 (24.4)

**Table 2:** Differences in optical parameters between malignant and benign pancreatic tissue.

	Malignant		Benign		*p*-value	
	Median	IQR	Median	IQR	40% BVF	10% BVF
BVF (%)	0.86	0.30-2.03	4.49	1.28-15.47	0.046	0.146
StO_2_ (%)	16.61	0.00-39.16	8.36	0.00-20.15	0.315	0.223
VD (µm)	6.04	2.66-14.40	12.5	3.92-68.38	0.382	0.947
BIL (µMol/L)	22.2	6.92-38.07	32.9	24.90-175.0	0.138	0.241
Mie amplitude	8.96	6.44-11.55	7.24	5.52-11.3	0.486	0.999
Mie slope	0.79	0.46-1.14	0.49	0.05-0.99	0.395	0.189
Rayleigh amplitude	0.05	0.00-0.07	0.02	0.00-0.05	0.295	0.527

Abbreviations: BVF: blood volume fraction; StO_2_: microvascular saturation; VD: blood vessel diameter; BIL: bilirubin concentration; IQR: interquartile range.

## Abbreviations

AUC: area under the curve
BIL: bilirubin concentration
BVF: blood volume fraction
CI: confidence interval
EUS: endoscopic ultrasound
FNA: fine needle aspiration
FNB: fine needle biopsy
IQR: interquartile range
NPV: negative predictive value
PDAC: pancreatic ductal adenocarcinoma
PPV: positive predictive value (BVF)
SD: standard deviation
SFR: single fiber reflectance
StO_2_: microvascular saturation
VD: vessel diameter
