# Supplementary material for: Single fiber reflectance spectroscopy for pancreatic cancer detection during endoscopic ultrasound guided fine needle biopsy: a prospective cohort study
Source: Int J Med Sci. 2022 Jan 1;19(2):205–12. doi: 10.7150/ijms.65364 (PMC8795801; doi:10.7150/ijms.65364)
Supplement: Supplementary file 1 — Supplementary figures and table. [file ijmsv19p0205s1.pdf]

Supplementary data

Table S1 Binomial logistic regression model

	**Odds ratio**	**95% confidence interval**	***p*-value**
BVF (%)	0.51	0.22-1.19	0.118
StO_2 (%)	1.10	0.99-1.23	0.090
BIL (µMol/L)	0.99	0.96-1.01	0.331
Serum BIL (µMol/L)	1.01	0.99-1.03	0.224

Abbreviations: BVF: blood volume fraction; StO_2: microvascular saturation; BIL: bilirubin concentration.

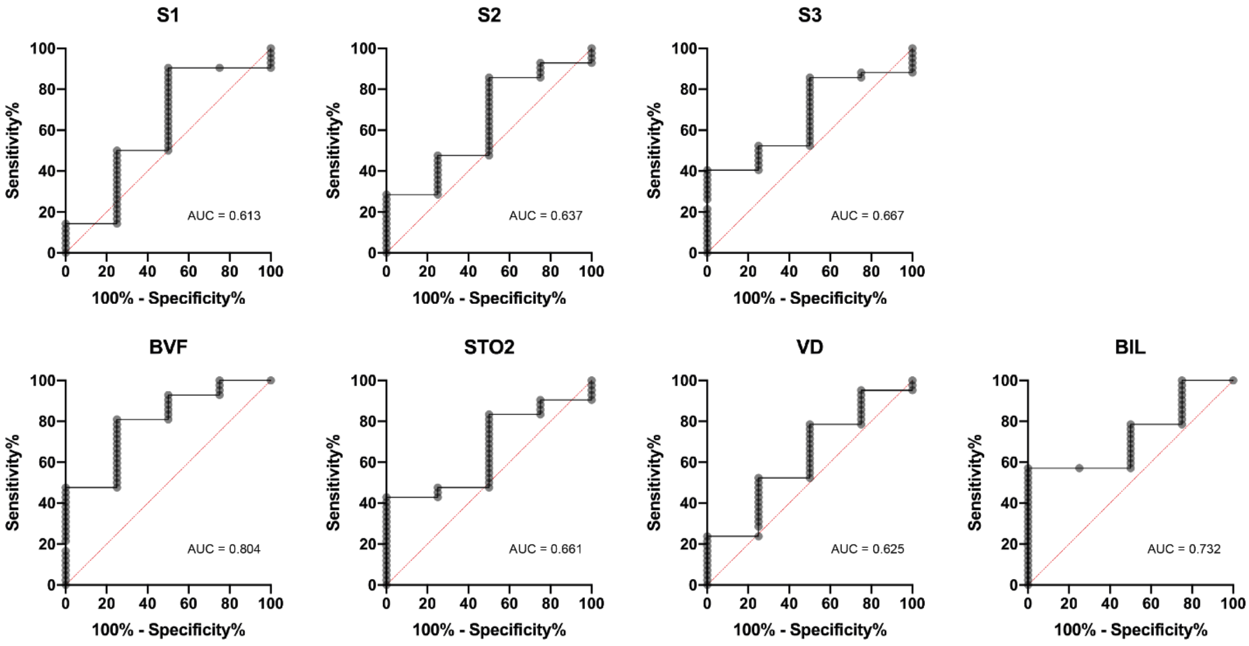

Figure S1. Overview on receiver-operating characteristic (ROC) curves of individual parameters after linear discriminant analysis.

Abbreviations: S1: Mie amplitude; S2: Mie slope; S3: Rayleigh amplitude; BVF: blood volume fraction; StO_2*: microvascular saturation; VD: blood vessel diameter; BIL: bilirubin concentration.*

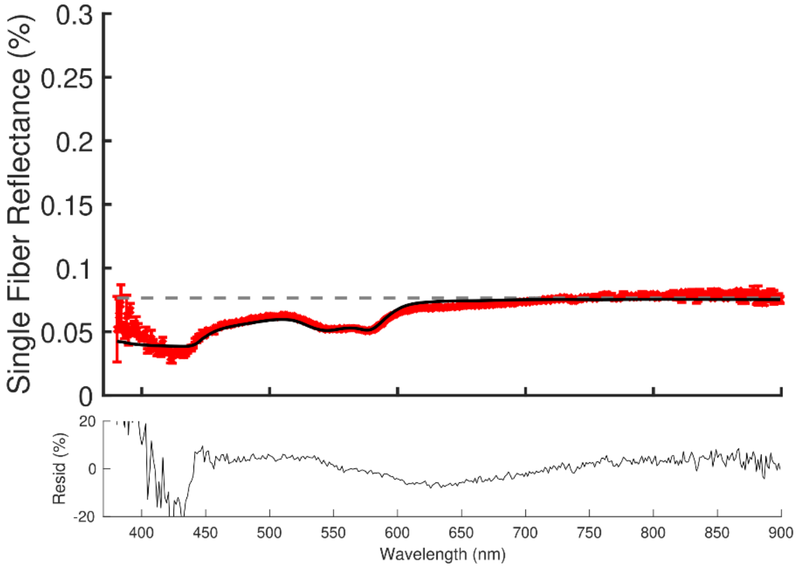

Figure S2. An example of reflectance spectra of normal pancreatic tissue. In two patients it was possible to perform measurements in normal pancreatic tissue (based on endoscopic ultrasound (EUS) view of the endoscopist) during the retraction of the fiber after a biopsy in a suspicious lesion. Due to the risk on EUS induced pancreatitis no biopsy has been taken in normal looking pancreatic tissue, so these measurement could not be histologically confirmed.
